# Circular RNA hsa_circ_0000467 Promotes the Development of Gastric Cancer by Competitively Binding to MicroRNA miR-326-3p

**DOI:** 10.1155/2020/4030826

**Published:** 2020-02-06

**Authors:** Wei Lie Mo, Jing Ting Jiang, Luo Zhang, Qi Cheng Lu, Jing Li, Wei Dong Gu, Yun Cheng, Hai Tao Wang

**Affiliations:** ^1^Department of Gastrointestinal Surgery, Third Affiliated Hospital of Soochow University, Changzhou, Jiangsu 213003, China; ^2^Jiangsu Engineering Research Center for Tumor Immunotherapy, Third Affiliated Hospital of Soochow University, Changzhou, Jiangsu 213003, China; ^3^Department of Gastrointestinal Surgery, Changzhou Seventh People's Hospital, Changzhou, Jiangsu 213011, China

## Abstract

Circular RNAs are a class of endogenous noncoding RNAs that play an important role in gene regulation. These RNAs are involved in the development and progression of various cancers, but their roles in gastric cancer have not yet been thoroughly elucidated. This study showed that hsa_circ_0000467 expression was higher in gastric cancer tissues than in corresponding adjacent tissues (*P* < 0.050) and that hsa_circ_0000467 expression levels were correlated with gastric cancer histological grade (*P* < 0.050). In addition, hsa_circ_0000467 was remarkably upregulated in gastric cancer cell lines (*P* < 0.001). Cell function experiments indicated that hsa_circ_0000467 downregulation decreased the proliferation and invasion ability of BGC-823 and SGC-7901 cells and the number of cells entering the G2/M phase. A direct binding interaction was detected between hsa_circ_0000467 and miR-326-3p by dual-luciferase reporter assays. In addition, the results showed that inhibition of miR-326-3p reversed the decreases in the proliferation and invasion of BGC-823 and SGC-7901 cells caused by hsa_circ_0000647 downregulation. Inhibition of miR-326-3p also decreased the number of cells entering the G2/M phase and the expression of cyclin D1. In conclusion, hsa_circ_0000467 plays a regulatory role in the development and progression of gastric cancer by regulating miR-326-3p, and this circRNA may be a potential diagnostic marker and therapeutic target of gastric cancer.

## 1. Introduction

Gastric cancer (GC) is a common digestive system tumor that occurs worldwide and is the second most common cause of cancer morbidity and mortality in China [1]. In 2015, approximately 498,000 people died of GC in China [2]. The 5-year survival rate of patients with early GC is more than 90%. However, most patients have lost the opportunity for effective surgery when they are diagnosed, meaning that their prognosis is poor. It is important to study the process of GC to find new potential molecular targets for GC therapy.

Circular RNAs (circRNAs) exist widely in humans. These RNAs are covalently closed-loop structures that do not have 5′ to 3′ polarity or a polyadenylation tail [3]. CircRNAs are stable in nature and not easily cleaved by Ribonuclease R [4]. CircRNAs can regulate gene expression, adsorb miRNA via a sponge action, regulate miRNA activity [5], and participate in the translation of proteins [6]. CircRNAs have been demonstrated to be critically involved in malignancies, such as liver cancer [7], bladder cancer [8], esophageal cancer [9], breast cancer [10], and prostate cancer [11].

MicroRNAs (miRNAs) are a common class of noncoding, single-stranded RNA molecules with a length of 19–25 nucleotides that can regulate the expression of corresponding mRNAs by targeting their three-prime untranslated region (3′-UTR) [12]. Some studies have shown that circRNAs can competitively bind to specific miRNAs to regulate gene expression and affect cancer development. Zeng et al. found that circHIPK3 could affect cells proliferation, migration, invasion, and induced apoptosis of colorectal cancer by targeting miR-7 [13]. It has been shown that circRNA-000284 promotes proliferation and invasion by regulating miR-506/Snail-2 in cervical cancer cells [14]. The regulatory function of circRNAs as “miRNA sponges” in GC remains generally unknown. Therefore, further research is needed.

In this study, we examined the expression level of hsa_circ_0000467 in GC tissues and corresponding adjacent tissues by qRT-PCR; we also detected its expression in GC cells and normal gastric mucosal cells. We confirmed a higher expression level of hsa_circ_0000467 in GC tissues, as well as in cell lines. At the same time, we evaluated the clinical significance of our findings. The effects of hsa_circ_0000467 downregulation on the proliferation, invasion, and cell cycle of GC cells were verified by CCK8 assays, Transwell assays, and flow cytometry. In addition, we explored the possible molecular mechanisms of hsa_circ_0000467 in promoting GC development by competitively binding to miR-326-3p through dual-luciferase reporter assays and rescue assays. Finally, we provided new ideas for potential new diagnostic and therapeutic targets of GC.

## 2. Materials and Methods

### 2.1. Clinical Samples

Cancerous tissues and corresponding adjacent tissues were collected from 30 patients with GC from October 2017 to January 2018 at the Third Affiliated Hospital of Soochow University. No patients received preoperative radiotherapy or chemotherapy, and all patients were confirmed by pathology as having gastric adenocarcinoma and classified according to TNM staging. GC specimens and corresponding normal stomach mucosa tissues were chopped and stored in liquid nitrogen until further use. This study was approved by the Ethics Review Committee of Soochow University (No. SUERC-GC-2017-048). Before the experiment, all patients signed written informed consent.

### 2.2. Cell Culture

The GES-1, BGC-823, and SGC-7901 cell lines were used in this study. All of the cells were obtained from the Chinese Academy of Sciences and Shanghai Institutes for Biological Sciences. The cells were cultured in RPMI 1640 medium (Gibco, NY, USA) containing 10% fetal bovine serum (FBS, Gibco, NY, USA). All cells in this medium were placed in 5% CO_2_ at 37°C. The cells were passaged every 2 to 3 days and were used for the experiments within 6 months.

### 2.3. RNA Isolation and Quantitative Real-Time PCR

Total RNA was extracted using Trizol reagent (Invitrogen, CA, USA). RNA concentrations were measured by Beckman Coulter, and each of the paired RNA samples was adjusted to the same concentration. A One-Step SYBR PrimeScript RT-PCR Kit II (Takara, Kusatsu, Japan) was used to conduct qRT-PCR assays for hsa_circ_0000467. A TaqMan MicroRNA Assays Kit (Applied Biosystems, CA, USA) was used to assay miR-326-3p. We used GAPDH and U6 as internal controls, and qRT-PCR assays were performed on an ABI 7500 Real-Time PCR System (Applied Biosystems, CA, USA). The relative gene expression of hsa_circ_0000467 and miR-326-3p in tissue specimens and cells was shown as the fold change (2^−ΔΔCT^). Each qRT-PCR analysis was repeated three times. All of the primers were synthesized by Ribobio, and the primer sequences are shown in Table 1.

### 2.4. RNA Interference and Transfection Assay

The GC cell lines BGC-823 and SGC-7901 were separately plated into four 60 mm culture dishes with 7.0 × 10^5^ cells in each dish and then transfected with Lipofectamine RNAi MAX (Thermo Fisher, CA, USA). The knockdown efficiency was examined by qRT-PCR using RNA extracted 48 h after transfection. GenePharma (Shanghai, China) synthesized the si-NC, si-hsa_circ_0000467, miR-326-3p inhibitor, and si-hsa_circ_0000467 + miR-326-3p inhibitor.

### 2.5. Cell Proliferation Assay

In this experiment, cells from different transfection groups were inoculated on 96-well plates. Based on a comprehensive evaluation of various factors, the number of cells used in each well was 2,000. Then, the cells were incubated at 37°C. At 0 h, 24 h, 48 h, and 72 h, and 10 *μ*l CCK8 solution (C0038, Beyotime, Shanghai, China) was added to the wells. After 1 h of incubation, the absorbance was measured at 450 nm by a microtiter plate reader (Molecular Devices, CA, USA). The final results were calculated as the average values measured three times under the same conditions. The GraphPad Prism5.0 software (La Jolla, USA) was used to plot the histograms.

### 2.6. Cell Invasion Assay

Different groups of BGC-823 and SGC-7901 cells were plated onto a Matrigel-coated membrane in the upper well of a 24-well Transwell insert and 600 *μ*l of DMEM (11965-118, Gibco, NY, USA) was added to the lower chamber (CLS3428-24EA, Corning, NY, USA). These cells were cultured in a 37°C, 5% CO_2_ incubator for 24 h. Finally, the Transwell chamber was rinsed, fixed in methanol, and stained with a 0.1% crystal violet solution (C0121, Beyotime, Shanghai, China), and the cells were observed under a microscope and photographed.

### 2.7. Cell Cycle Analysis

BGC-823 and SGC-7901 cells were collected by centrifugation at low speed for five minutes, followed by adequate washing with phosphate buffer, and then, the cells were fixed with 70% ethanol, incubated for 12 h at 4°C in an ice bath, and stained with Ribonuclease A (ST576, Beyotime, Shanghai, China) and PI (P4170, Sigma Aldrich, USA) staining buffer. The cells were suspended in staining solution (P4170, Sigma Aldrich, MS, USA), and the cell cycle was analyzed by flow cytometry (BD, Biosciences, NJ, USA). The Modfit software (Verity Software House, GA, USA) provided the estimation of the percentage of cells in G0/G1, S, and G2/M phases of the cycle.

### 2.8. Bioinformatics Analysis

The online target predicting database Circular RNA Interactome (https://circinteractome.nia.nih.gov) was used to predict potential downstream miRNAs of hsa_circ_0000467. Then, qRT-PCR assays were used to detect the expression of these miRNAs when hsa_circ_0000467 was downregulated.

### 2.9. Dual-Luciferase Reporter Assay

The sequences of hsa_circ_0000467 containing the predicted miR-326-3p binding sites were cloned into the psiCHECK-2 vector (Promega, WI, USA) and were named psiCHECK-hsa_circ_0000467-wild type (WT) and psiCHECK-hsa_circ_0000467-mutant (Mut). BGC-823 cells were cotransfected with miR-326-3p mimics and psiCHECK-hsa_circ_0000467-WT or psiCHECK-hsa_circ_0000467-Mut. Next, a dual-luciferase reporter assay system (Promega, WI, USA) was used to detect luciferase activity after transfection for 24 h. Finally, the results were recorded.

### 2.10. Western Blot Analysis

BGC-823 and SGC-7901 cells were lysed with RIPA lysis buffer (P0013C, Beyotime, Shanghai, China) containing the protease inhibitor PMSF (Sigma, MS, USA). Protein quantification was performed using a Bradford protein assay kit (P0006, Beyotime, Shanghai, China). Next, polyacrylamide gel electrophoresis was used to separate equal amounts of protein, and 30 *μ*g of protein for each sample was then transferred to polyvinylidene fluoride membranes (Millipore, USA). The membranes were blocked with skim milk powder and incubated with primary antibody for 12 h at 4°C. The primary antibodies used for western blotting were rabbit anti-cyclin D1 (1 : 2000, Ab134175, Abcam, LON, UK), rabbit anti-c-MYC (1 : 3000, Ab32072, Abcam LON, UK), and rabbit anti-HRP-GAPDH (1 : 5000, KC-5G5, Aksomics, Shanghai, China). Then, the blots were immunostained with a secondary antibody for 1 h to 2 h. The second antibody was goat anti-rabbit (1 : 7000, A0545, Sigma, MS, USA). The cells were then washed three times with TBST, subjected to chemiluminescence, and finally imaged with a gel imaging system. The relative expression of the protein was analyzed by densitometry analysis using Quantity One Analysis Software (Bio-Rad, CA, USA).

### 2.11. Statistical Analysis

The collected data were analyzed and processed using SPSS 22.0 (IBM Corporation, Chicago, IL, USA). We used GraphPad Prism 5.0 software (La Jolla, USA) to generate the figures. The results are described as the mean ± standard deviation (SD), and the count data are expressed as percentages. For analyzing differences in results, an independent sample *t*-test method was selected, and a variance test was carried out. The difference level was *P* < 0.05.

## 3. Results

### 3.1. Hsa_circ_0000467 Was Highly Expressed in GC Tissues and Cells and Was Markedly Associated with the GC Histological Grade

**Figure 1:**
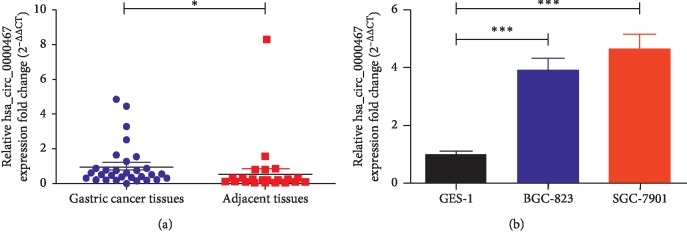
Expression of hsa_circ_0000467 in GC tissues and cell lines. (a) The relative hsa_circ_0000467 expression in tissues was detected by qRT-PCR. (b) Hsa_circ_0000467 expression in cell lines was detected by qRT-PCR. The results are shown as the fold change (2^−ΔΔCT^) and the mean ± SD from three times experiments. ^*∗*^*P* < 0.05, ^*∗∗∗*^*P* < 0.001.

We performed a circRNA microarray assay using paired samples from 10 patients (H1710082 AS-CR-005 Human Circular RNA Microarray v2) and detected >10,000 circRNAs differentially expressed between the GC tissues and their corresponding adjacent tissues. These circRNAs were ranked according to the fold changes in expression between the groups, and the top 15 upregulated circRNAs are presented in Table 2. We selected hsa_circ_0000467 as our research focus, and the other circRNAs were studied by other members of our research team. To study the role of has_circ_0000647 in GC, its expression levels in human GC tissues and normal gastric mucosa tissues were analyzed by qRT-PCR. The expression results of the two groups were compared. The results showed that has_circ_0000647 was significantly higher in GC tissues than in normal tissues (Figure 1(a)). On this basis, the expression of hsa_circ_0000467 in two GC cell lines (BGC-823, SGC-7901) and one normal gastric mucosal cell line (GES-1) was analyzed, and the results were compared. The qRT-PCR results showed that hsa_circ_0000467 was upregulated in GC cell lines (Figure 1(b)). Moreover, to further explore the correlation between the clinicopathological features of GC patients and the hsa_circ_0000467 expression level, 30 GC patients were divided into high hsa_circ_0000467 expression group and low hsa_circ_0000467 expression group according to the median value, and the number of patients in the two groups was equal. The results demonstrated that the upregulation of hsa_circ_0000467 was related to the worse grade of GC (*P*=0.0224). However, there was no correlation between its expression level and other clinicopathological features (Table 3).

### 3.2. Silencing hsa_circ_0000467 Reduced the Proliferation and Invasion of GC Cells and Affected the Cell Cycle

**Figure 2:**
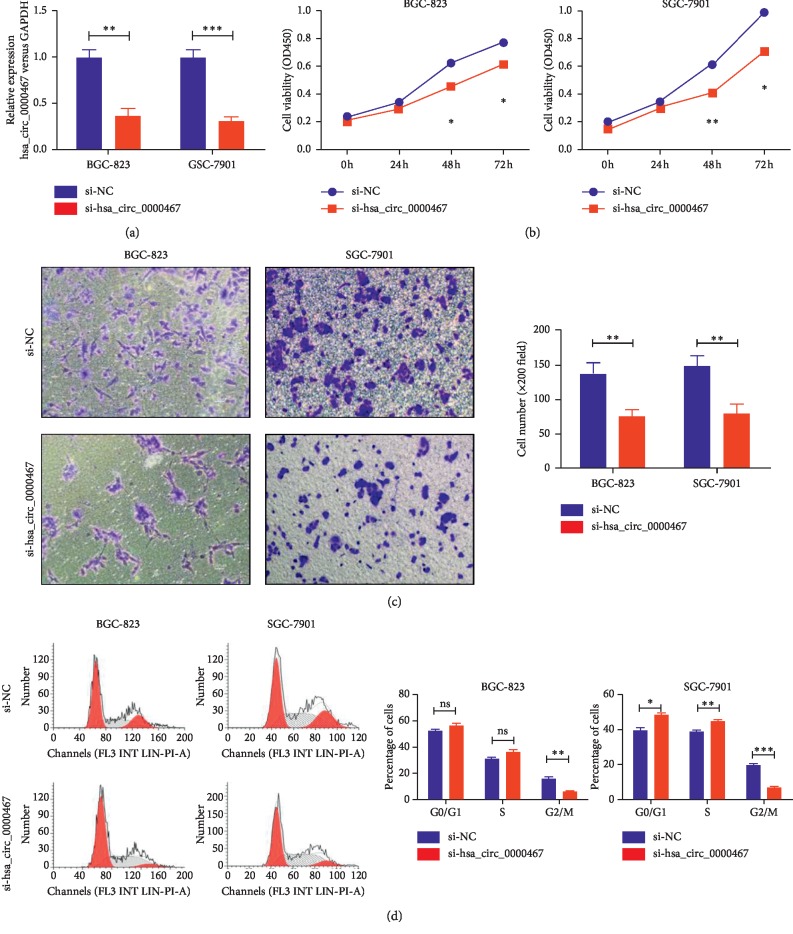
Effect of hsa_circ_0000467 silencing on GC proliferation, invasion ability, and cell cycle. (a) The interference efficacy of siRNA targeting hsa_circ_0000467 was measured by qRT-PCR. (b) The proliferation activity of BGC-823 and SGC-7901 cells transfected with si-NC or si-hsa_circ_0000467 was detected by CCK8 assays. (c) The invasion ability of BGC-823 and SGC-7901 cells transfected with si-NC or si-hsa_circ_0000467 was detected by Transwell assays. (d) The cell cycle of BGC-823 and SGC-7901 cells transfected with si-NC or si-hsa_circ_0000467 was detected by flow cytometry. The results are represented as the means ± SD of at least three times. ns: not significant, ^*∗*^*P* < 0.05, ^*∗∗*^*P* < 0.01, ^*∗∗∗*^*P* < 0.001.

In an attempt to study the biological function of the target circRNA in GC, we designed a small interfering RNA (siRNA) to silence hsa_circ_0000467 in BGC-823 and SGC-7901 cells. qRT-PCR was used to verify the transfection efficiency (Figure 2(a)). We then examined the effect of hsa_circ_0000467 on cell proliferation and invasion by CCK8 and Transwell assays. The results showed that hsa_circ_0000467 downregulation inhibited the proliferation and invasion ability of these two cell lines (Figures 2(b) and 2(c)). Cell cycle assays showed that the silencing of hsa_circ_0000467 inhibited BGC-823 cells entering the G2/M phase. Similarly, it arrested SGC-7901 cells in the G0/G1 phase, and the number of cells entering the G2/M phase was significantly reduced (Figure 2(d)).

### 3.3. Hsa_circ_0000467 Has Binding Sites with miR-326-3p

**Figure 3:**
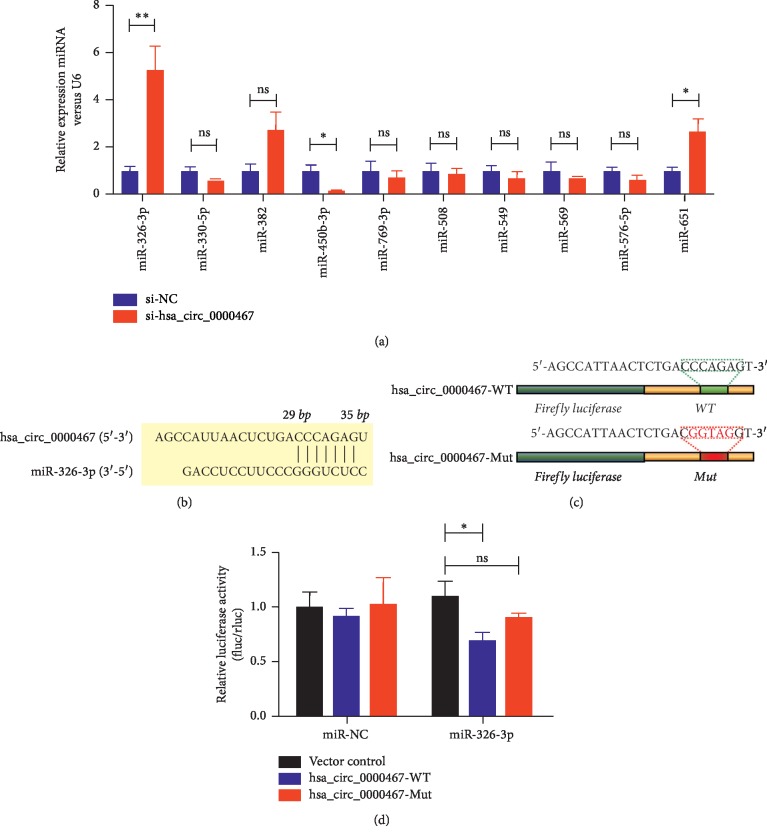
Hsa_circ_0000467 has binding sites with miR-326-3p. (a) Differential miRNA expression in gastric cancer cells in which hsa_circ_0000467 was decreased was detected by qRT-PCR. (b) Schematic model showing the putative binding sites for miR-326-3p and hsa_circ_0000467. (c) Construction of dual-luciferase reporter plasmids containing the WT or Mut hsa_circ_0000467 sequence. (d) The luciferase activities of the blank controls or miR-326-3p mimics and the WT or Mut hsa_circ_0000467 cotransfected BGC-823 cells were determined by luciferase reporter assays. ns: not significant, ^*∗*^*P* < 0.05, ^*∗∗*^*P* < 0.01.

To explore the mechanism of hsa_circ_0000467 in the process of tumorigenesis, we explored the target miRNAs of hsa_circ_0000467. We determined the sequence of hsa_circ_0000467 through an online target prediction database (Circular RNA Interactome) and identified the miRNAs that were most likely to bind to hsa_circ_0000467; then, we verified the targets by qRT-PCR assays. The results showed that under decreased hsa_circ_0000467 conditions, the expression level of miR-326-3p in GC was the highest (Figure 3(a)).  Figure 3(b) shows the predicted binding sites of miR-326-3p and hsa_circ_0000467. To determine whether hsa_circ_0000467 could sponge miR-326-3p, we constructed a dual-luciferase reporter plasmid containing the WT or Mut hsa_circ_0000467 sequence (Figure 3(c)). The results showed that the overexpressing miR-326-3p inhibited the luciferase activity of the WT reporter for hsa_circ_0000467 but not the Mut reporter for hsa_circ_0000467 (Figure 3(d)). These data indicated a highly efficient interaction between hsa_circ_0000467 and miR-326-3p via the binding sites and provided direct evidence of miR-326-3p sponging by hsa_circ_0000467.

### 3.4. Hsa_circ_0000467 Affects the Biological Function of GC by Sponging miR-326-3p

**Figure 4:**
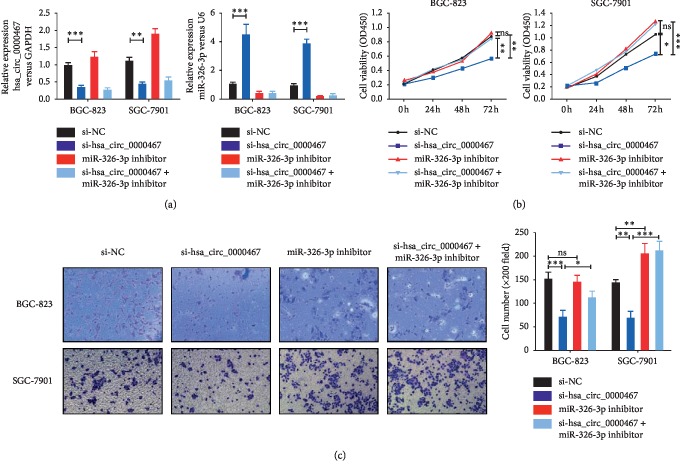
Changes in the proliferation and invasion of BGC-823 and SGC-7901 cells caused by low has_circ_0000647 expression could be reversed by knocking down miR-326-3p. (a) Expression of hsa_circ_0000467 and miR-326-3p in different groups of BGC-823 and SGC-7901 cells was detected by qRT-PCR. (b) The proliferation activities of different groups of these two cell lines were measured by CCK8 assays. (c) The invasion ability of different groups of these two cell lines was detected by Transwell assays. The data are presented as the mean ± SD. ns: not significant, ^*∗*^*P* < 0.05, ^*∗∗*^*P* < 0.01, ^*∗∗∗*^*P* < 0.001.

**Figure 5:**
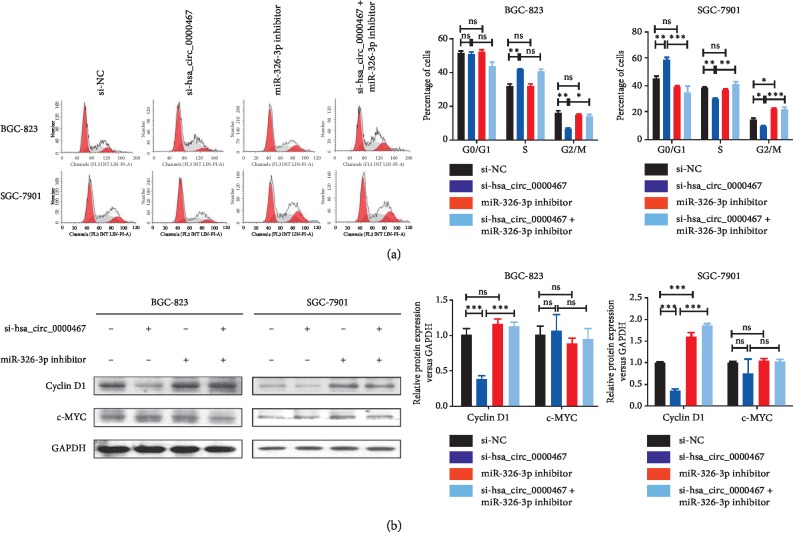
Changes in the cell cycle and cyclin D1 expression of GC cells induced by low has_circ_0000647 expression could be reversed by knocking down miR-326-3p. (a) Cell cycle distribution of different groups of BGC-823 and SGC-7901 cells was detected by flow cytometry. (b) The expression levels of cyclin D1 and c-MYC in different groups of these two cell lines were analyzed by western blot assay. The data are presented as the mean ± SD. ns: not significant, ^*∗*^*P* < 0.05, ^*∗∗*^*P* < 0.01, ^*∗∗∗*^*P* < 0.001.

To verify whether the target circRNA regulated the proliferation, invasion, and cell cycle of GC cells through miR-326-3p, we performed rescue experiments with hsa_circ_0000467 and miR-326-3p. The GC cell lines BGC-823 and SGC-7901 were used to create four different transfection groups: si-NC, si-hsa_circ_0000467, miR-326-3p inhibitor, and si-hsa_circ_0000467 + miR-326-3p inhibitor. The transfection efficiency was verified by qRT-PCR (Figure 4(a)). We found that the amount of miR-326-3p in BGC-823 and SGC-7901 cells increased significantly after knocking down hsa_circ_0000467. The CCK8 assay results showed that downregulation of miR-326-3p suppressed the decrease in proliferation ability caused by hsa_circ_0000467 knockdown (Figure 4(b)). Similarly, downregulation of hsa_circ_0000467 and miR-326-3p could eliminate the decreased invasive capacity caused by knocking down only hsa_circ_0000467 (Figure 4(c)). Furthermore, the cell cycle assays indicated that knockdown of hsa_circ_0000467 caused cell cycle arrest at the G0/G1 phase, and the number of cells entering the G2/M stage was reduced. However, these effects could be reversed when miR-326-3p was also downregulated (Figure 5(a)). In addition, western blot analysis showed that inhibition of the target circRNA decreased the expression of cyclin D1 in these two cell lines, while inhibition of miR-326-3p reversed this effect. However, the expression of c-MYC in these cells was not affected by the knockdown of hsa_circ_0000467 and miR-326-3p (Figure 5(b)).

## 4. Discussion

GC is a common digestive tract tumor. The incidence and mortality of GC are second to only lung cancer in China [2]. Although the 5-year survival rate of early GC after radical surgery and related adjuvant treatment can reach more than 90%, most cancers are middle or late stage when diagnosed because the symptoms of early GC are not obvious; thus, these patients have missed their opportunity for surgery. Even when surgery is performed, recurrence and mortality are still high. Therefore, we chose to focus on novel gene regulatory factors, circRNAs, which are a type of endogenous noncoding RNA widely distributed in the human body. Compared with traditional linear RNAs, the structure of cyclic RNAs is more complex and has obvious changes. The structure is a covalently closed ring with no polarity or polyadenylated tail [15]. CircRNAs have a stable nature, are highly conserved, and exhibit specific tissue expression. CircRNAs have functions such as regulating gene expression [16] and participating in protein translation [6].

We selected hsa_circ_0000467 as a research object due to the results of a microarray assay analysis. Subsequent qRT-PCR results showed that this target circRNA was highly expressed in GC tissues and cells. When we knocked down it, the proliferation and invasion ability of BGC-823 and SGC-7901 cells decreased, and the number of cells entering the G2/M stage was reduced significantly. These results indicated that hsa_circ_0000467 could affect the biological function of GC.

In recent years, there have been many studies on the role of miRNAs in cancer. It has been found that various abnormally expressed miRNAs are associated with GC; for example, aberrant expression of microRNA-31 may inhibit the proliferation of GC cells and induce apoptosis at an early stage [17]. MiR-204 inhibits the proliferation of GC cells by targeting CKS1B, CXCL1, and GPRC5A [18]. Many circRNAs contain miRNA response elements that bind to miRNAs. Zeng et al. found that circHIPK3 promoted the development of colorectal cancer by binding miR-7 [13]. Xie et al. found that circular RNA BCRC-3 suppressed bladder cancer proliferation through the miR-182-5p/p27 axis [19]. Based on this property of circRNAs and miRNAs, we predicted that miRNA could interact with hsa_circ_0000467. Finally, we found that miR-326-3p and hsa_circ_0000467 have binding sites in GC cells, and miR-326-3p mimics could inhibit the luciferase activity of WT hsa_circ_0000467, suggesting that hsa_circ_0000467 acted as a sponge of miR-326-3p in GC.

MiR-326 is a newly discovered miRNA. This RNA affects the expression of various cytokines and transcription factor activity by regulating the corresponding target genes. For this reason, miR-326-3p could participate in the development of GC. Ji et al. identified that miR-326 inhibited GC cell growth by downregulating NOB1 [20]. Li et al. demonstrated that downregulation of miR-326 was associated with poor prognosis and promoted GC growth [21]. To further verify the relationship between hsa_circ_0000467 and miR-326-3p in GC, we performed rescue experiments using different groups of BGC-823 and SGC-7901 cells. We found that knockdown of miR-326-3p could reverse the decrease in proliferation and invasion ability caused by knocking down hsa_circ_0000467. Similarly, knocking down hsa_circ_0000467 reduced the proportion of BGC-823 and SGC-7901 cells entering the G2/M phase, while downregulating hsa_circ_0000467 and miR-326-3p inhibited this effect.

Cyclin D1 and c-MYC are cyclins and proto-oncogenes involved in the development of GC. A variety of molecules can affect the biological function of GC by regulating cyclin D1 and c-MYC. For example, lycorine inhibits SGC-7901 cell proliferation mainly via reducing the expression level of cyclin D1, and the experimental results confirm this [22]. Similarly, microRNA-623 targeting of cyclin D1 also makes GC tumor cells more sensitive to 5-fluorouracil, thus improving the therapeutic effect of drugs [23]. GC mesenchymal stem cells can also promote the expression of PD-L1 through the STAT3/mTOR-c-Myc axis [24]. Therefore, we performed western blot assays to observe the expression of cyclin D1 and c-MYC in different groups. We found that inhibition of hsa_circ_0000467 reduced the expression of cyclin D1 in BGC-823 and SGC-7901 cells, and inhibition of miR-326-3p reversed this effect. However, the downregulation of hsa_circ_0000467 and miR-326-3p did not affect the expression of c-MYC.

Due to the short duration of the study, we did not compare hsa_circ_0000467 expression levels and patient survival rates. Further research will result in a better understanding of hsa_circ_0000467 and may provide more ideas for the diagnosis and treatment of GC.

In conclusion, the results of this paper show that hsa_circ_0000467 is highly expressed in GC and plays a regulatory role in promoting the development of GC. In addition, hsa_circ_0000467 affects the proliferation, invasion, and cell cycle of GC by sponging miR-326-3p. In the future, hsa_circ_0000467 might be used as a marker for the diagnosis and therapy of GC.

## Figures and Tables

**Table 1:** Oligonucleotide sequences for this study.

Name	Sequence
hsa_circ_0000467-F	5′-ACACAATGGGACTTAAAAATGCGA-3′
hsa_circ_0000467-R	5′-ACAGATCATCTTTCACATCAGTCT-3′
GAPDH-F	5′-CACCATCTTCCAGGAGCGAG-3′
GAPDH-R	5′-AAATGAGCCCCAGCCTTCTC-3′
miR-326-3p-F	5′-ACACTCCAGCTGGGCCTCTGGGCCCT-3′
miR-326-3p-R	5′-CTCAACTGGTGTCGTGGAGTCGGCAATTCAGTTGAGCTGGAGGA-3′
U6-F	5′-CTCGCTTCGGCAGCACA-3′
U6-R	5′-AACGCTTCACGAATTTGCGT-3′
si-NC	5′-GCCUCCGGCUUCGCACCUCU-3′
si-hsa_circ_0000467	5′-AUGUGCUCGUCUUUUAUUAU-3′
miR-326 inhibitor	5′-CCUCUGGGCCCUUCCUCCAG-3′

**Table 2:** Biological information regarding the top 15 upregulated circRNAs.

Alias^a^	FC (abs)	*P* value	circRNA type	Chr	Gene symbol
hsa_circ_0003893	4.1497811	0.001908727	Exonic	chr15	CEMIP
hsa_circ_0034537	3.869808	0.000285534	Exonic	chr15	THBS1
hsa_circ_0044556	3.5869766	0.001010661	Exonic	chr17	COL1A1
hsa_circ_0058230	3.5639838	0.013816053	Exonic	chr2	PNKD
hsa_circ_0004104	3.5493467	0.010071999	Exonic	chr5	SPARC
hsa_circ_0058097	3.5450916	0.000161903	Exonic	chr2	FN1
hsa_circ_0092303	3.4657038	0.024196064	Intronic	chr19	CACTIN
hsa_circ_0078715	3.4266263	0.00195079	Exonic	chr6	THBS2
hsa_circ_0002447	3.3414571	2.55292E-06	Exonic	chr2	COL6A3
hsa_circ_0008223	3.3061326	0.013097578	Exonic	chr16	XPO6
hsa_circ_0022382	3.189447	0.000190467	Exonic	chr11	FADS2
hsa_circ_0081069	3.1113818	0.00108704	Exonic	chr7	COL1A2
hsa_circ_0000467	3.0778227	0.000107203	Exonic	chr13	SKA3
hsa_circ_0007333	3.0338617	0.021394251	Exonic	chr2	PAX3
hsa_circ_0051239	3.0103916	0.031807734	Exonic	chr19	ATP5SL

^a^Alias refers to the circRNA ID in circBase. FC: fold changes; abs: absolute value; Chr: chromosome.

**Table 3:** Correlation between hsa_circ_0000467 expression and clinicopathological features of gastric cancer.

Clinicopathological features	Patients (*n* = 30)	hsa_circ_0000467 expression^a^		*P* value
		High group	Low group	
Gender				0.6903
Male	21	10 (33.3%)	11 (36.7%)	
Female	9	5 (16.7%)	4 (13.3%)	
Age (year)				0.6660
≥70	7	4 (13.3%)	3 (10.0%)	
<70	23	11 (36.7%)	12 (40.0%)	
Tumor size (cm)				0.7125
≥5	13	7 (23.3%)	6 (20.0%)	
<5	7	8 (26.7%)	9 (30.0%)	
Histological grade				0.0224^*∗*^
Well	3	0 (0.0%)	3 (10.0%)	
Moderately	12	4 (13.3%)	8 (26.7%)	
Poor	15	11 (36.7%)	4 (13.3%)	
TNM stage				0.1953
I and II	7	2 (6.7%)	5 (16.7%)	
III and IV	23	13 (43.3%)	10 (33.3%)	
Pathological T category				0.0674
T_1_	0	0 (0.0%)	0 (0.0%)	
T_2_	1	0 (0.0%)	1 (3.3%)	
T_3_	24	10 (33.3%)	14 (46.7%)	
T_4_	5	4 (13.3%)	1 (3.3%)	
Lymphatic node metastasis				0.9720
N_0_	7	3 (10.0%)	4 (13.3%)	
N_1_	2	1 (3.3%)	1 (3.3%)	
N_2_	11	6 (20.0%)	5 (16.7%)	
N_3_	10	5 (16.7%)	5 (16.7%)	

TNM: tumor node metastasis. Significant difference: ^*∗*^*P* < 0.05. ^a^Using median hsa_circ_0000467 values as cutoff.

## Data Availability

The data used to support the findings of this study are included within the article.
