# Correction to “Circular RNA hsa_circ_0000467 Promotes the Development of Gastric Cancer by Competitively Binding to MicroRNA miR‐326‐3p”

**DOI:** 10.1155/bmri/9797352

**Published:** 2026-07-28

W. L. Mo, J. T. Jiang, L. Zhang, et al. “Circular RNA hsa_circ_0000467 Promotes the Development of Gastric Cancer by Competitively Binding to MicroRNA miR‐326‐3p,” *BioMed Research International*, 2020, 4030826, https://doi.org/10.1155/2020/4030826

In the article, there is an error in Figure 2c, where the si‐NC/BGC‐823 panel in Figure 2c appears to be identical to the siNC panel in Figure 2b of [1], by many of the same authors. The corrected Figure 2 is shown below, in which the si‐has_circ_0000467/BGC‐823 has also been updated by the authors for consistency.

**Figure 2:**
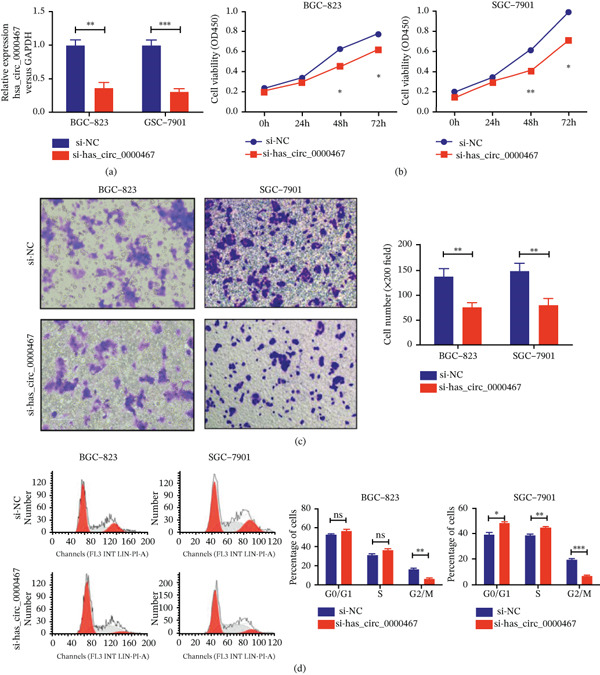
Effect of hsa_circ_0000467 silencing on GC proliferation, invasion ability, and cell cycle. (a) The interference efficacy of siRNA targeting hsa_circ_0000467 was measured by qRT‐PCR. (b) The proliferation activity of BGC‐823 and SGC‐7901 cells transfected with si‐NC or si‐hsa_circ_0000467 was detected by CCK8 assays. (c) The invasion ability of BGC‐823 and SGC‐7901 cells transfected with si‐NC or si‐hsa_circ_0000467 was detected by Transwell assays. (d) The cell cycle of BGC‐823 and SGC‐7901 cells transfected with si‐NC or si‐hsa_circ_0000467 was detected by flow cytometry. The results are represented as the means ± SD of at least three times. ns, not significant; ∗*p* < 0.05; ∗∗*p* < 0.01; ∗∗∗*p* < 0.001.

Moreover, during our investigation, overlapping regions were identified in Figure 4c between the SGC‐7901/miR‐326‐3p inhibitor panel and the SGC‐7901/si‐hsa_circ_0000467+miR‐326‐3p inhibitor panel. The authors have clarified that this error occurred due to mislabeling during the image selection process for the Transwell invasion assays. The correct Figure 4 is shown below:

**Figure 4:**
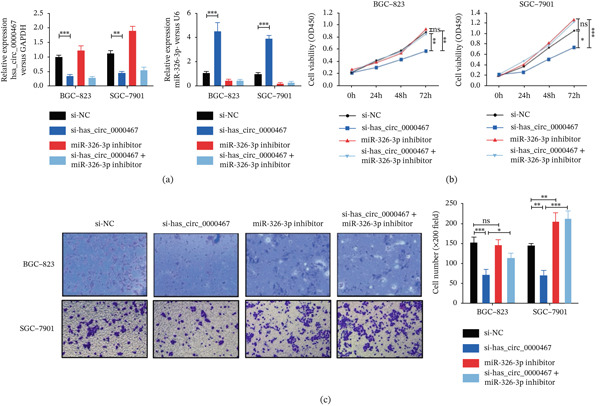
Changes in the proliferation and invasion of BGC‐823 and SGC‐7901 cells caused by low has_circ_0000647 expression could be reversed by knocking down miR‐326‐3p. (a) Expression of hsa_circ_0000467 and miR‐326‐3p in different groups of BGC‐823 and SGC‐7901 cells was detected by qRT‐PCR. (b) The proliferation activities of different groups of these two cell lines were measured by CCK8 assays. (c) The invasion ability of different groups of these two cell lines was detected by Transwell assays. The data are presented as the mean ± SD. ns, not significant; ∗*p* < 0.05; ∗∗*p* < 0.01; ∗∗∗*p* < 0.001.

The authors apologize for these errors.
